# RNA-binding protein with serine-rich domain 1 regulates microsatellite instability of uterine corpus endometrial adenocarcinoma

**DOI:** 10.6061/clinics/2021/e3318

**Published:** 2021-11-10

**Authors:** Xiaojuan Liu, Hui Ma, Lisha Ma, Kun Li, Yanhua Kang

**Affiliations:** Department of Gynecology and Obstetrics, The First Affiliated Hospital of Hebei North University, Qiaoxi District, Zhangjiakou City 075000, Hebei Province, P.R. China.

**Keywords:** RNPS1, UCEC, MMR, Proliferation

## Abstract

**OBJECTIVE:**

To determine the role of RNA-binding protein with serine-rich domain 1 (*RNPS1*) in uterine corpus endometrial carcinoma (UCEC), the role of *RNPS1* knockdown in UCEC development *in vitro* and *in vivo*, and the relationship between *RNPS1* and mismatch repair (MMR) in UCEC.

**METHODS:**

We predicted the potential function of *RNPS1* using bioinformatics systems. The expression of *RNPS1* in tissues and cell lines was analyzed by western blotting and immunohistochemistry. The expression of *RNPS1* in MMR was assessed using bioinformatics and western blotting. The proliferation and apoptosis of UCEC cells were assessed under *RNPS1* knockdown conditions, and *RNPS1* regulation in MMR was detected by suppressing Notch signaling. Associations between *RNPS1* and gene mutations in UCEC and prognosis were analyzed.

**RESULTS:**

The *RNPS1* level was higher in UCEC tumors than in normal tissues and tumors or RL952 cells. Prognostic outcomes were worse when UCEC showed abundant *RNPS1* expression. Lentiviral *RNPS1* knockdown weakened tumor cell proliferation and suppressed biomarker expression, reduced the tumor volume, promoted apoptosis *in vitro* and *in vivo*, and inhibited UCEC development. Increased MutS homolog 2 (*MSH2*) and MutS homolog 6 (*MSH6*) levels in MMR after *RNPS1* knockdown were reversed by inhibiting Notch signaling. Furthermore, *RNPS1* was associated with mutations in *NAA11*, *C2orf57*, *NUPR1*, and other genes involved in UCEC prognosis.

**CONCLUSION:**

*RNPS1* may regulate the expression levels of *MSH2* and *MSH6* in MMR, enhancing the proliferation, development, and prognosis of UCEC through a Notch signaling pathway in UCEC. Our study offers a new method and strategy for delaying UCEC development through modulating MMR.

## INTRODUCTION

Uterine corpus endometrial carcinoma (UCEC) is a common gynecological carcinoma with a high recurrence rate (1). Endometrioid adenocarcinoma (EC) is the most common pathological manifestation of UCEC, with proliferative endometrial tumor cells showing glandular complexity and cellular pleomorphism (2). The risk of recurrence is intermediate or high in patients with EC after radical hysterectomy combined with pelvic and para-aortic lymphadenectomy, a considerable challenge for clinics (3). Although evolving medical technologies have led to long-term declines in mortality due to EC, the mechanisms and treatment of EC require further exploration.

DNA mismatch repair (MMR) protein deficiency is a result of microsatellite instability (MSI), a hallmark of EC (4,5), providing the biological relevance and potential utility of the modal classification of EC and increasing the complexity of the genomic instability underlying tumorigenesis (6). As MMR regulation may be a prognostic factor for patients with advanced UCEC (7,8), an MMR regulator for treating UCEC needs to be identified.

RNA-binding protein with serine-rich domain 1 (*RNPS1*) belongs to the mRNA nuclear export and mRNA surveillance post-splicing multiprotein complex (9) involved in lung squamous cell and ovarian adenocarcinoma (10,11). However, the mechanisms underlying the role of *RNPS1* in MMR in UCEC remain unclear.

The Notch signaling pathway is implicated in many cancers, including in the maintenance of cancer stem cells, metabolism, survival, drug resistance, epithelial-mesenchymal transition, and genomic instability (12). The Notch signaling pathway may enhance the invasive properties of EC, indicating that it may serve as a promising target for the treatment of this malignancy (13).

The present study aimed to determine the role of *RNPS1* in UCEC. We used an *RNPS1* knockdown lentivirus (sh-RNPS1) to regulate MMR progression through the Notch signaling pathway and tumor progression in UCEC. The present findings indicate that *RNPS1* regulates MMR in UCEC. These results may provide novel strategies for UCEC therapy *via* MMR-associated mechanisms.

## MATERIALS AND METHODS

### UCEC specimens from patients

Sixteen patients pathologically diagnosed with moderately differentiated (G2), type I, MMR-deficient EC were selected at the Department of Pathology of the First Affiliated Hospital of Hebei North University from May 2019 to May 2021, with adjacent para-carcinoma tissues serving as controls. All patients provided written informed consent to participate and the use of their samples was approved by the ethics committee of this study.

### Bioinformatics analysis

The expression of *RNPS1* in various types of cancers was analyzed using TIMER2.0 (http://timer.cistrome.org/). Gene set enrichment analysis (GSEA) data were analyzed as previously described (14). The survival of patients with UCEC showing low and high *RNPS1* expression was assayed using the Kaplan-Meier plotter (http://kmplot.com/analysis/). Pan-cancer was analyzed as previously described (15). Genes related to mutation and prognosis were analyzed using target gene assays (https://www.mutarget.com/analysis?type=target).

### Cell lines and cell culture

Normal endometrial (NECs; CP-H058), KLE (CL-0133), RL952 (CL-0197), and Ishikawa (CL-0283) cells (all from Procell, Wuhan, China) and ECC-1 cells (BS-C163325, BinSuiBio, Shanghai, China) were maintained and cultured as previously described (16).

### Animals

Healthy male Balb/c mice (Chongqing Tengxin Biotechnology Co., Ltd., Chongqing, China) were housed under specific pathogen-free conditions. The mice were managed by the Care and Use of Laboratory Animals (NIH Publication No. 85-23, revised 1996), and the Ethics Committee of the First Affiliated Hospital of the Hebei North University approved the experimental design (protocol No: 2019ECHNU033).

### Antibodies

The following antibodies were purchased from the respective suppliers: anti-RNPS1 (ab79233), anti-Ki-67 (ab15580), anti-CEA (ab207718), anti-CA199 (ab3982), anti-CA153 (ab109185), anti-HE4 (ab200828), anti-Bcl-2 (ab182858), anti-Bax (ab182733), anti-MLH1 (ab92312), anti-cleaved caspase-3 (ab2302), anti-MSH2 (ab212188), anti-MSH6 (ab92471), and anti-PMS2 (ab110638) (all from Abcam, Cambridge, USA) and anti-β-actin (M01263-2; Boster, Wuhan, China). The secondary antibodies used were anti-rabbit IgG (AS014) and anti-mouse IgG (H+L) (AS003), both from ABclonal (Wuhan, China), and IMR-1A (HY-100431A; MedChemExpress, Dallas, TX, USA).

### RNPS1-knockdown lentivirus administration

An *RNPS1*-knockdown (sh-RNPS1) lentivirus and a control lentivirus (Table 1) were designed and chemically synthesized (GenePharma Corporation, Shanghai, China) and stored at -80°C. The cells were transduced with the lentiviruses, as previously described (17).

The mice were randomly assigned to the following groups (n=6 per group): control (con), untreated RL952 cells, or mice injected with RL952 cells; sh-RNPS1, RL952 cells transduced with sh-RNPS1 lentivirus or mice injected with RL952 cells infected with sh-RNPS1; lentivirus control (LC)-shRNPS1, lentivirus cells treated with *RNPS1* control lentivirus, or mice injected with lentivirus cells incubated with *RNPS1* control lentivirus; and IMR-1A+sh-RNPS1, RL952 cells incubated with sh-RNPS1 lentivirus, or mice injected with RL952 cells incubated with sh-RNPS1 lentivirus or IMR-1A (10 μg *in vitro* or 10 mg/kg *in vivo*).

### UCEC mouse model

The mice were subcutaneously injected with RL952 cells 5 days later, as previously described (18). The mice were sacrificed when they rapidly lost >20% of their weight and showed signs of deteriorating health, such as hunching, dehydration, and labored breathing due to the metastatic burden.

### Western blotting

Proteins (50 μg) from each sample were resolved by 12% SDS-polyacrylamide gel electrophoresis and blotted onto nitrocellulose membranes as previously described (19).

### Immunohistochemistry (IHC)

Tissue sections (4-μm-thick) were incubated with primary antibodies against *RNPS1* and Ki-67 at 4°C overnight, followed by incubation with a secondary antibody, as previously described (20).

### MTT assay

After stirring the samples for 30 min, they were passed through a filter with pores (diameter, 0.22 µm), and then stored at 4°C, as previously described (21).

### Statistical analysis

Data are expressed as the means±standard deviations and were statistically analyzed by one-way or two-way ANOVA using SPSS version 19.0 (SPSS Inc., Chicago, IL, USA). The Holm-Sidak test was used for multiple comparisons. Statistical significance was set at *p*<0.05.

## RESULTS

### Identifying the potential function of RNPS1 in UCEC using bioinformatics analysis

**Figure 1:**
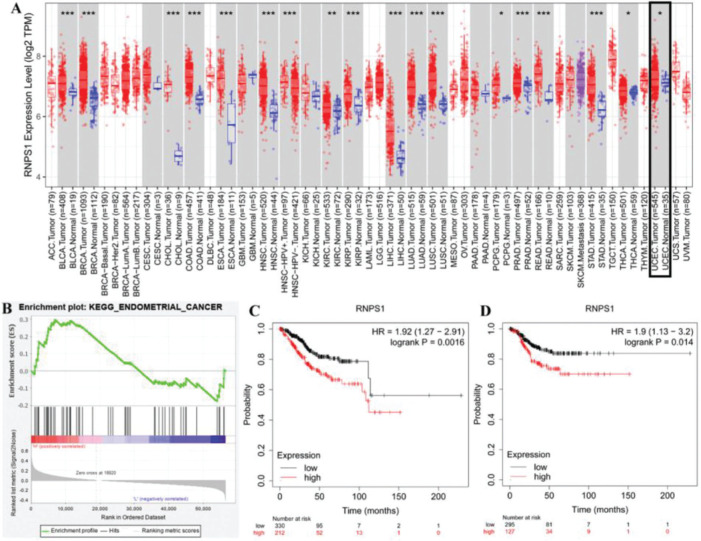
(A) Assessment of *RNPS1* expression in cancers. (B) GSEA of the correlation between *RNPS1* and UCEC. (C) OS and (D) RFS analyses of the role of *RNPS1* in UCEC patients.

Figure 1 shows the predicted role of *RNPS1* in UCEC detected by TIMER2.0, GSEA, and a Kaplan-Meier plotter. Figure 1A shows that the expression of *RNPS1*, as determined by TIMER2.0, was higher in UCEC tumors than in normal tissues (*p*<0.05). The GSEA findings (Figure 1B) showed that *RNPS1* was positively correlated with UCEC progression (*p*<0.05). Analysis of the overall survival (OS) and recurrence-free survival (RFS) indicated that the prognostic outcomes of patients with UCEC and high *RNPS1* expression (Figure 1C and D) were worse (*p*<0.05).

### Different levels of RNPS1 in tissues or cell lines of UCEC

**Figure 2:**
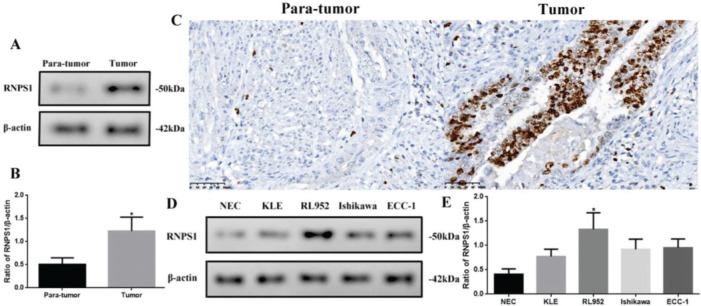
(A) *RNPS1* expression, (B) quantitation of the *RNPS1* expression levels from the western blots, and (C) IHC staining analysis for *RNPS1* in para-tumor and tumor tissues (×400). (D) *RNPS1* expression in NEC, KLE, RL952, Ishikawa, and ECC-1 cells. (E) Quantitation of the *RNPS1* expression levels in cells. Protein levels were normalized to those of β-actin. (n=6, *p<0.05: tumor *vs.* para-tumor or RL952 *vs.* other cells).

As *RNPS1* might be involved in UCEC, we analyzed the differences in *RNPS1* expression among UCEC tissues or cell lines (Figure 2). The *RNPS1* level was significantly higher in tumors than in para-tumor tissues (*p*<0.05, Figure 2A and B). Similar to the IHC results (Figure 2C), *RNPS1* was localized in the nucleus. In addition, *RNPS1* expression was significantly higher in RL952 cells than in other cells (*p*<0.05; Figure 2D and E). Therefore, we used RL952 as the UCEC model cell line associated with *RNPS1* for further investigation.

### Development of UCEC *in vitro* was regulated after RNPS1 knockdown

**Figure 3:**
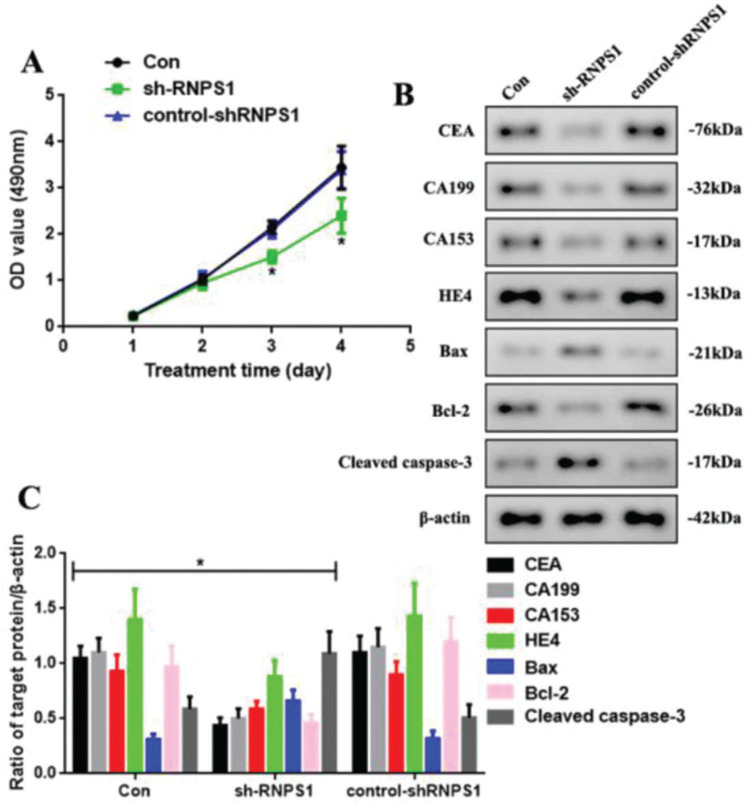
(A) Proliferation assays of cells from the Con, sh-RNPS1. and control-shRNPS1 groups at 4 days. (B) Western blots for the analysis of the levels of CEA, CA199, CA153, HE4, Bax, Bcl-2. and cleaved caspase-3. (C) Quantitation of the levels of CEA, CA199, CA153, HE4, Bcl-2, Bax, and cleaved caspase-3. (n=6, *p<0.05: sh-RNPS1 *vs.* other groups).

We knocked down RNPS1 using the lentivirus sh-RNPS1 to verify the role of RNPS1 in UCEC and its correlation with the previously obtained results (Figure 3). The control, sh-RNPS1, and control-shRNPS1 RL952 cells proliferated (Figure 3A). However, the optical density of the samples, as detected by MTT, decreased at 3 and 4 days in the sh-RNPS1 group (*p*<0.05). We measured the levels of apoptotic and tumor biomarkers of UCEC to determine their regulation after RNPS1 knockdown (Figure 3B). The levels of CEA, CA199, CA153, HE4, and Bcl-2 were reduced, whereas the activities of Bax and cleaved caspase-3 were induced in the sh-RNPS1 group (*p*<0.05, Figure 3C).

### Development of UCEC *in vivo* was regulated after RNPS1 knockdown

**Figure 4:**
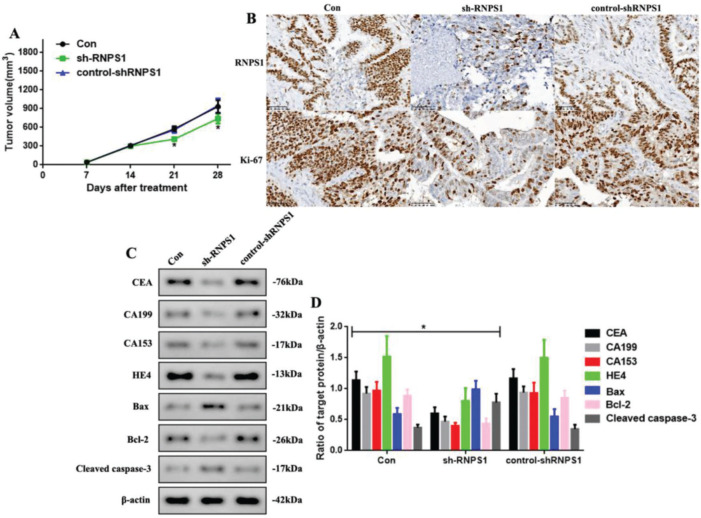
(A) Tumor volume in mice from the Con, sh-RNPS1, and control-shRNPS1 groups at 28 days. (B) IHC analysis for detecting the *RNPS1* levels and Ki-67 index. (C) Western blots for the analysis of the levels of CEA, CA199, CA153, HE4, Bcl-2, Bax, and cleaved caspase-3, (D) Quantitation of the levels of CEA, CA199, CA153, HE4, Bax, Bcl-2, and cleaved caspase-3. (n=6, *p<0.05: sh-RNPS1 *vs.* other groups).

As *RNPS1* knockdown regulated the progress of UCEC *in vitro*, we examined whether this also occurred *in vivo* (Figure 4). We found that the tumor volumes of the sh-RNPS1 mice were significantly decreased at 21 and 28 days (*p*<0.05, Figure 4A), and the level of *RNPS1* and the proliferation index, Ki-67, were reduced (Figure 4B). We assessed the levels of apoptotic and tumor biomarkers in UCECs (Figure 4C). The levels of CEA, CA199, CA153, HE4, and Bcl-2 decreased, while those of Bax and cleaved caspase-3 increased (*p*<0.05, Figure 4F).

### Bioinformatic analyses of the functions of RNPS1 in MSI and MMR in UCEC by pan-cancer, GSEA, and correlation analysis

**Figure 5:**
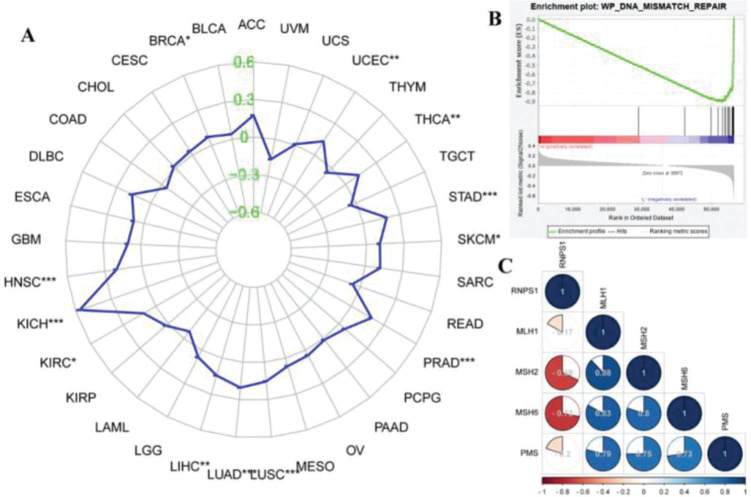
(A) Pan-cancer assays of MSI in different cancers. (B) GSEA of the correlation between *RNPS1* and MMR in UCEC. (C) Analysis of the correlation of *RNPS1* with *MSH1, MSH2, MSH6,* and *PMS2* in UCEC.

Figure 5A shows the functions of *RNPS1* and MSI in different types of cancer. We found that *RNPS1* was positively associated with MSI in the UCEC. The GSEA results showed that *RNPS1* was negatively for MMR (Figure 5B). Correlation analysis verified that *RNPS1* was negatively correlated with the MMR markers *MSH2* and *MSH6* but positively correlated with *MSH1* and *PMS2* in UCEC (Figure 5B).

### MMR marker levels were increased by RNPS1 knockdown *in vivo*

**Figure 6:**
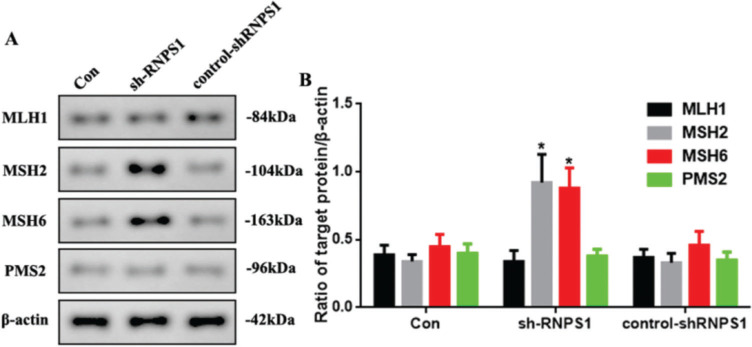
(A) Western blots for the analysis of the *MSH1, MSH2, MSH6*, and *PMS2* expression levels *in vivo*, (B) Quantitation of the *MSH1, MSH2, MSH6,* and *PMS2* expression levels. (n=6, *p<0.05: sh-RNPS1 *vs.* other groups).

As previous predictions have indicated that the *RNPS1* gene is associated with MSI and MMR in UCEC, we examined MMR marker levels *in vivo*. Figure 6A shows the increased expression of *MSH2* and *MSH6* in the sh-RNPS1 group (*p*<0.05) compared with the other groups (Figure 6B).

### Mismatch repair was regulated by RNPS1 knockdown through Notch signaling pathway *in vivo*

**Figure 7:**
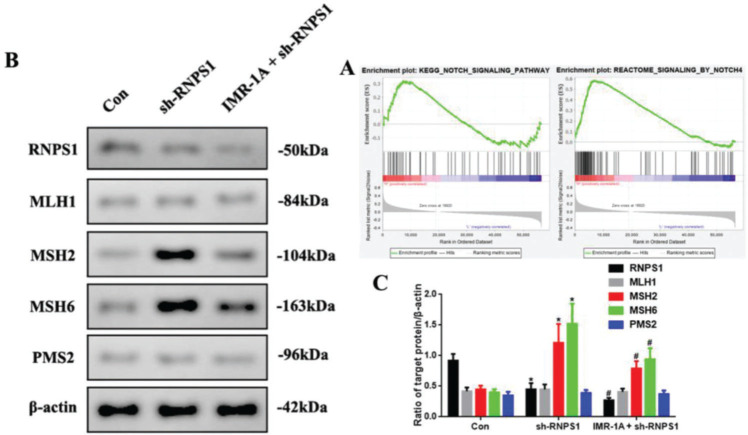
(A) GSEA of the correlation between *RNPS1* and Notch or Notch4 signaling pathway in UCEC. (B) Western blot assay for the analysis of the *RNPS1, MSH1, MSH2, MSH6,* and *PMS2* expression levels *in vivo*. (B) Quantitation of *RNPS1, MSH1, MSH2, MSH6,* and *PMS2* expression levels. (n=6, *p<0.05: sh-RNPS1 *vs.* Con; ^#^ *p*<0.05: IMR-1A+sh-RNPS1 *vs.* sh-RNPS1).

The GSEA findings showed that *RNPS1* was positively for Notch, especially, Notch4 signaling (Figure 7A). To confirm this, we blocked Notch expression using an inhibitor of Mastermind Recruitment-1A (IMR-1A). Figure 7B shows that IMR-1A decreased the levels of *MSH2* and *MSH6* after *RNPS1* knockdown (*p*<0.05) compared with the case in the sh-RNPS1 group (Figure 7C); notably, IMR-1A reduced the *RNPS1* levels.

### Further bioinformatics analysis of RNPS1 in patients with UCEC using target gene assays

**Figure S1:**
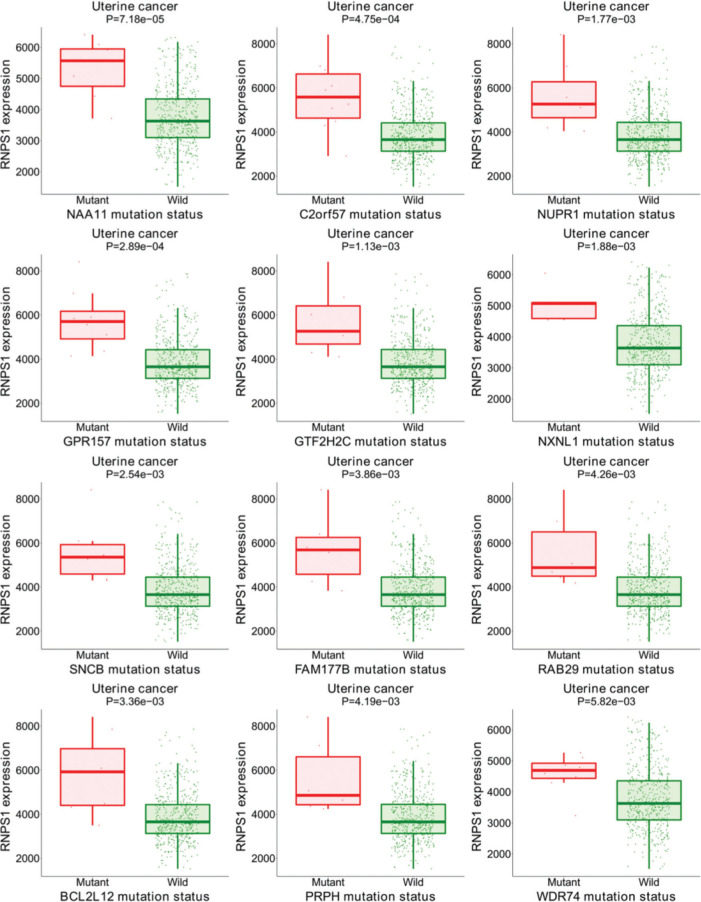
(A) Expression of *RNPS1* in UCEC patients with mutant or wild-type *NAA11*, *C2orf57*, *NUPR1*, *GPR157*, *GTF2H2C*, *NXNL1*, *SNCB*, *FAM177B*, *RAB29*, *BCL2L12*, *PRPH*, and *WDR74*.

Figure S1 shows the results of the target gene assays of *RNPS1* in UCEC tissues. Mutations were found in the group with higher levels of *RNPS1, NAA11, C2orf57, NUPR1, GPR157, GTF2H2C, NXNL1, SNCB, FAM177B, RAB29, BCL2L12, PRPH,* and *WDR74*. These results suggest that *RNPS1* is associated with these gene mutations and participates in the prognosis of UCEC.

## DISCUSSION

Endometrioid EC is the most common uterine malignancy, with a mortality rate of approximately 20%; it is the most prevalent gynecological malignancy in western and developed countries (22,23). The mortality rates of endometrioid EC are increasing, indicating the need for more effective diagnostic and treatment strategies (24). MMR deficiency due to the loss of MMR protein expression, a hotspot mutation in the POLE exonuclease domain, and a nonspecific molecular profile may be prognostic factors for patients with advanced UCEC (7,8,25).

A recent whole-genome analysis found that *RNPS1* regulates carcinogenesis, especially, in EC (26). As little is known about *RNPS1*, it should be a hot topic in oncological research. In the present study, bioinformatics and molecular biological methods were applied to determine the functions of *RNPS1* in UCEC. Higher levels of *RNPS1* are expressed in UCEC tumors than in normal tissues; the prognostic outcomes of patients with UCEC were poor, and abundant expression of the *RNPS1* isoform were observed. These results showed that *RNPS1* may be an oncogene involved in the prognosis of UCEC. To verify this issue, we compared the levels of the *RNPS1* protein in various tissues and cell lines. The results showed that *RNPS1* expression was increased in EC tissue and RL952 cells, consistent with the findings of the bioinformatics analyses.

Apoptosis is a programmed cell death that does not elicit an inflammatory response (27). Knocking down RNPS1 weakened tumor cell proliferation and biomarkers, reduced tumor volume, increased apoptosis *in vitro* and *in vivo*, and inhibited UCEC development in our study. These results showed that RNPS1 could regulate apoptosis and tumor progression in UCEC and were consistent with the findings of the bioinformatics analyses.

We further explored the mechanism underlying the action of RNPS1 in EC using bioinformatics methods and found that RNPS1 correlated positively with MSI and negatively with MMR proteins, especially, *MSH2* and *MSH6*. Western blotting confirmed the bioinformatic findings of a relationship between RNPS1 and MMR, as the *RNPS1* knockdown lentivirus improved the expression of *MSH2* and *MSH6* but not *MSH1* and *PMS2*. We plan to report this issue, but some methodological deficiencies resulting from limited laboratory conditions prevent more rigorous and advanced experimentation. Other scholars should investigate these findings further.

Clonal diversity was due to the accumulation of mutations in genes of diverse pathways, such as Notch and MMR pathways (28). Notch signaling can provide the first mechanistic example of altered glycosylation in tumor cells with MSI (29). Our GSEA findings showed that *RNPS1* correlated positively with Notch signaling, especially, the Notch4 signaling pathway in UCEC. We investigated this aspect by blocking Notch signaling using IMR-1A. The results showed that IMR-1A decreased *MSH2* and *MSH6* levels after lentivirus-based *RNPS1* knockdown. Furthermore, IMR-1A reduced the *RNPS1* levels, suggesting that Notch is an upstream factor for *RNPS1*. However, as a specific Notch4 inhibitor is not yet available, we could only inhibit Notch signaling. The present results showed that *RNPS1* may be a downstream target factor of Notch; therefore, the Notch4 signaling pathway may regulate MMR in UCEC.

Bioinformatic analyses of *RNPS1* in patients with UCEC using the target gene system showed that *RNPS1* may be associated with mutations in *NAA11, C2orf57, NUPR1, GPR157, GTF2H2C, NXNL1, SNCB, FAM177B, RAB29, BCL2L12, PRPH,* and *WDR74*, participating in UCEC prognosis. These data await confirmation in future investigations and will be the focus of our subsequent studies.

We found that *RNPS1* knockdown significantly activated apoptosis and inhibited EC development and *MSH2* and *MSH6* expression *via* the Notch signaling pathway. Overall, our findings offer a new strategy for treating UCEC.

## AUTHOR CONTRIBUTIONS

Liu X designed the study and manuscript drafting. Ma H and Ma L analyzed the data. Li K wrote and revised the manuscript. Kang Y polished the first manuscript drafting and designed the methodology.

## Figures and Tables

**Table 1:** Sequences of RNPS1 lentiviruses.

Name	Sequence
sh-RNPS1-421	5′-taataaaaagtccagcacta-3′
sh-RNPS1-601	5′-tcggtccagctcgacttcca-3′
sh-RNPS1-1262	5′-cgctccagctccaactcctc-3′
Negative Control	5′-ataccccacccagctcagtt-3′
Control-shRNPS1	5′-ttgtcacttttctagccaaa-3′
